# Increased systemic exposures of artemether and dihydroartemisinin in infants under 5 kg with uncomplicated *Plasmodium falciparum* malaria treated with artemether-lumefantrine (Coartem®)

**DOI:** 10.1186/s12936-015-0682-7

**Published:** 2015-04-15

**Authors:** Alfred B Tiono, Halidou Tinto, Maroufou J Alao, Martin Meremikwu, Antoinette Tshefu, Bernhards Ogutu, Alphonse Ouedraogo, Moussa Lingani, Marc Cousin, Gilbert Lefèvre, Jay Prakash Jain, Stephan Duparc, Kamal Hamed

**Affiliations:** Centre National de Recherche et de Formation sur le Paludisme, Ministère de la Santé, 01 BP 2208 Ouagadougou, Burkina Faso; Unité de Recherche Clinique de Nanoro (IRSS-CRUN), BP 218, Ouagadougou, CMS11 Burkina Faso; Service de Pédiatrie, Hôpital de la Mère et de l’Enfant Lagune, Cotonou, 01 BP 107 Benin; Institute of Tropical Disease Research and Prevention, University of Calabar Teaching Hospital, Calabar, PMB 1278 Nigeria; Kinshasa School of Public Health, University of Kinshasa, 11850 Kinshasa, Democratic Republic of Congo; Centre for Clinical Research, Kenya Medical Research Institute, Nairobi, Kenya; Novartis Pharma AG, CH-4002 Basel, Switzerland; Novartis Healthcare Private Limited, Hyderabad, India; Medicines for Malaria Venture (MMV), Route de Pré-Bois 20, 1215 Meyrin, Switzerland; Novartis Pharmaceuticals Corporation, One Health Plaza, East Hanover, NJ 07936-1080 USA

**Keywords:** Artemether-lumefantrine, Dispersible, Efficacy, Infants, Pharmacokinetics, Safety, <5 kg body weight

## Abstract

**Background:**

Artemether-lumefantrine (AL) dispersible formulation was developed for the treatment of uncomplicated *Plasmodium falciparum* malaria in infants and children weighing 5 to <35 kg. However, there are no clinical studies with artemisinin-based combination therapy in infants <5 kg.

**Methods:**

This multicentre, open-label, single-arm study evaluated the efficacy, safety and pharmacokinetics of AL dispersible in infants aged >28 days and <5 kg of body weight, who were treated with one AL dispersible tablet (20 mg artemether/120 mg lumefantrine) given twice-daily for three days and followed up for six weeks (core follow-up) and at 12 months of age (long-term follow-up).

**Results:**

A total of 20 patients were enrolled and completed the six-week core study follow-up. In the per protocol population, PCR-corrected cure rate at days 28 and 42 was 100% (95% CI: 79.4, 100). AL dispersible was well tolerated with reported adverse events of mild to moderate severity. Pharmacokinetic data showed that lumefantrine levels were similar, however, artemether and dihydroartemisinin levels were on average two- to three-fold greater than historical values in infants and children ≥5 kg.

**Conclusions:**

A three-day regimen of AL dispersible formulation was efficacious and generally well tolerated in infants weighing <5 kg with uncomplicated *P. falciparum* malaria, but artemether and dihydroartemisinin exposures could not be supported by the preclinical safety margins for neurotoxicity. Hence, dosing recommendations cannot be made in infants <5 kg as implications for toxicity are unknown.

**Trial Registration:**

Clinicaltrials.gov NCT01619878.

## Background

Artemether-lumefantrine ((AL) Coartem®/Riamet®, Novartis Pharma AG, Basel, Switzerland) is the first fixed-dose artemisinin-based combination therapy (ACT) to be prequalified by the World Health Organization for the treatment of uncomplicated *Plasmodium falciparum* malaria in adults and children weighing ≥5 kg [1-3]. The paediatric formulation of AL (AL dispersible), tailored to the needs of infants and children weighing 5 to <35 kg, is a sweet tasting and easy-to-administer formulation. Since its launch in 2009, >250 million AL dispersible treatments have been delivered to over 50 malaria-endemic countries. AL dispersible was tested in a large multicentre trial in 899 children and was found to be as efficacious as crushed tablet, with a similar safety profile [4]. The efficacy of AL dispersible was similar across different body weight dosing groups [5], and the formulation was easier to use and more acceptable, as compared with the crushed tablet in children [6,7].

While much is known about malaria in infants and children weighing ≥5 kg, there are no clinical trials or national guidelines for treatment of infants <5 kg. The current treatment for this subgroup is oral quinine, which is associated with a poor safety profile [8]. Other anti-malarial treatments are frequently used off-label, on the basis of recommended dosing schedules for older infants and children [9,10]. Although some studies have reported ACT use in this subgroup [9], no ACT has been approved for use in infants <5 kg, with the exception of artesunate-amodiaquine, which is registered for the treatment of infants ≥4.5 kg. Thus, there is an unmet medical need in infants <5 kg with uncomplicated *P. falciparum* malaria. This study aimed to evaluate the efficacy, safety and pharmacokinetics (PK) of AL dispersible following treatment with a three-day regimen in infants <5 kg with uncomplicated *P. falciparum* malaria.

## Methods

### Patients

Male and female infants aged >28 days and weighing <5 kg, with microscopically confirmed acute uncomplicated *P. falciparum* malaria, including asexual *P. falciparum* parasitaemia >1,000 and <100,000 parasites/μL, were included. Patients were recruited from three healthcare facilities in two countries in Africa, one in Benin and two in Burkina Faso. Patients with severe malaria or general danger signs (i.e., signs of a critical condition or severely deteriorated general condition) based on the Integrated Management for Childhood Illnesses (IMCI) criteria for sick infants were excluded from the study. The trial protocol was approved by an Independent Ethics Committee (IEC) and the Institutional Review Board (IRB) at each study centre (a Swiss IEC and two local IRBs). Signed informed consent was obtained from parent or legal guardian before any study-related procedure. The study was conducted in accordance with the International Conference on Harmonization Guidelines for Good Clinical Practice, Declaration of Helsinki, and local regulations of each participating country. This trial is registered with with ClinicalTrials.gov number NCT01619878.

### Study design

This multicentre, open-label, single-arm study was planned to be conducted in two sequential cohorts of infants <5 kg; cohort 1 in infants >28 days old and cohort 2 in neonates ≤28 days of age. Based on an interim analysis of cohort 1 results after the six-week core follow-up period, cohort 2 was not initiated as recommended by a data monitoring committee. The study was terminated after all patients from cohort 1 completed their long-term follow-up.

Patients were admitted to the hospital during a three-day treatment phase and followed up until day 42 (week 6) after treatment, with regular assessments for efficacy and safety during the six weeks. A long-term follow-up was conducted at 12 months of age to assess neurodevelopmental status. Patients in cohort 1 received directly observed treatment of AL dispersible tablet (20 mg artemether/120 mg lumefantrine) following a regimen of one dispersible tablet twice daily for three consecutive days. The consumption of food or drink (mother’s or formula milk) was recommended after dose to enhance lumefantrine absorption. In the event of vomiting within an hour of dosing, a repeat dose was administered and a maximum of two doses were replaced throughout the entire treatment phase.

Patients who developed severe malaria or early treatment failure signs were to receive rescue therapy as per local treatment guidelines. Presence of parasitaemia on day 7, irrespective of clinical state, and vomiting of replacement dose within one hour warranted rescue therapy.

### Study assessments

Clinical and parasitological examinations were performed on days 0, 1, 2, 3, 7, 28, and 42. Giemsa-stained thick and thin smears were examined locally at each visit using a light binocular microscope fitted with an oil immersion lens for assessment of parasitaemia. Blood smears were also systematically read at a central facility. Blood samples for molecular diagnostics (PCR-based methods) were taken at baseline, days 14, 28 and 42, and at any unscheduled visit when reappearance of parasitaemia was confirmed microscopically.

Physical examination was performed on days 2, 3, 4, 7, 14, 28, and 42, or at the time of withdrawal. Vital signs including systolic and diastolic blood pressure (measured with Dynascope DS-7100, Fukuda Denshi Co., Tokyo, Japan, with an appropriately sized cuff), temperature, and pulse rate were measured. Blood samples were collected and analysed locally for haematology and blood chemistry tests.

Safety monitoring included recording of all adverse events (AEs) and serious AEs (SAEs). Neurodevelopmental status was assessed using an age-appropriate scale (Shoklo neurodevelopmental scale) at 12 months of age [11].

Blood samples (250 μL each) were drawn at predefined timepoints: one and two hours after the first AL dose for measurement of artemether and its active metabolite dihydroartemisinin (DHA) in plasma; six hours after the fifth and sixth doses, 24 hours after the sixth dose, and on day 7 for lumefantrine measurement. Artemether and DHA were analysed using reversed-phase HPLC with MS/MS detection and the limit of quantification (LOQ) was 5 ng/mL. Lumefantrine was determined using reversed-phase HPLC with MS/MS detection and the LOQ was 50 ng/L.

### Statistical analysis and sample size

It was aimed to enroll 40 patients to obtain a sample size of at least 30 patients evaluable for the primary endpoint (at least 15 evaluable patients in each cohort). Based on an assumed PCR-corrected day 28 cure rate of 95% and a sample size of 30 evaluable patients, the study had 80% power to result in an estimate of cure rate associated with a lower exact 95% confidence limit >77%. For secondary efficacy variables, Exact Pearson-Clopper two-sided 95% confidence limits were constructed for dichotomous outcome variables (cure rates at days 3, 7, 14, 28, and 42). Incidence of gametocyte carriage over time was summarized by baseline gametocyte carriage status.

Descriptive summary statistics were presented for values and change from baseline to each follow-up. For PK exposure to artemether, DHA and lumefantrine, descriptive statistics of concentrations at discrete timepoints were presented. However, no formal PK analysis was performed due to limited number of sample points.

The infant neurodevelopmental scale included the total scores for motor milestones (section A), coordination (section B), tone (section C), behaviours (section D), and the sum of all sections together. All patients who received at least one dose of the study drug and had confirmed falciparum malaria at baseline were included in the full analysis set (FAS). The evaluable patient set (EPS) comprised patients from the FAS who had a known 28-day cure status, i.e., those who completed the study at least until day 28, or were considered failures before day 28 for reasons other than premature withdrawal. Patients from the EPS weighing <5 kg at baseline, who took at least five of six doses of the study drug, had baseline asexual parasite counts >1,000 and <100,000 parasites/μL based on central microscopy reading, and did not take any other medication with anti-malarial effect (excluding rescue medication) up to day 28 were included in the per protocol set (PPS).

### Outcome measures

PCR-corrected parasitological cure rate at day 28 was the primary efficacy outcome defined as the proportion of patients clearing asexual parasites within seven days of initiating the study treatment without recrudescence before or at day 28, corrected for re-infection using PCR. The secondary efficacy outcomes included PCR-corrected parasitological cure rates at days 14 and 42 and uncorrected cure rates at days 3, 7, 14, 28, and 42.

Artemether, DHA and lumefantrine concentrations were determined at discrete timepoints during and after treatment with AL dispersible. Safety parameters included incidence of AEs, SAEs and routine safety laboratory assessments.

## Results

**Figure 1:**
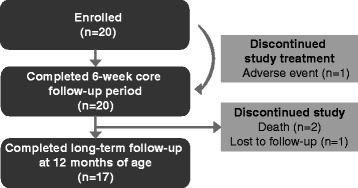
Subject disposition.

A total of 20 infants were recruited into the study and completed the six-week core follow-up. One patient discontinued study treatment because of vomiting but remained in the study. No patients received rescue therapy. A total of 17 patients completed the long-term follow-up visit at 12 months of age (Figure 1). Cohort 2 was not initiated as recommended by the data monitoring committee due to increased artemether and dihydroartemisinin exposures observed during an interim analysis and the potential risk of neurotoxicity based on preclinical data.

The median age (range) was 82 (37-214) days and median body weight (range) was 4.8 (2.6-4.9) kg. There was an equal number of male and female patients, and all patients were of African origin. The patients’ demographic and baseline characteristics are shown in Table 1.

**Table 1:** **Demographics and baseline characteristics**

**Parameters**	**N = 20**
Age (days)	
>28 days, n (%)	20 (100)
Mean ± SD	99.1 ± 51.8
Median	82.0
Min-max	37-214
Sex, n (%)	
Female	10 (50)
Race, n (%)	
Black	20 (100)
Weight, (g)	
Mean ± SD	4,333.8 ± 733.8
Median	4,765.0
Min-max	2,570-4,925
Height, (cm)	
Mean ± SD	56.6 ± 4.1
Median	56.0
Min-max	48-65
Parasite species, n (%)	
*Plasmodium falciparum*	20 (100)
*Plasmodium ovale*	1 (5)
Others	0 (0)
*Plasmodium falciparum* asexual forms (/μL)	n = 20
Mean ± SD	13,783.6 ± 19,214.5
Median	7,273
Min-max	1,014-84,147
*Plasmodium falciparum* density categories, n (%)	
1,000-5,000/μL	8 (40)
>5,000	12 (60)
*Plasmodium falciparum* gametocytes (/μL)	n = 3
Mean ± SD	606.7 ± 640.8
Median	481
Min-max	38-1,301
Body temperature (°C)	n = 20
Mean ± SD	37.5 ± 0.9
Median	37.2
Min-max	36.0-39.0

Max, maximum; Min, minimum; SD, standard deviation.

**Figure 2:**
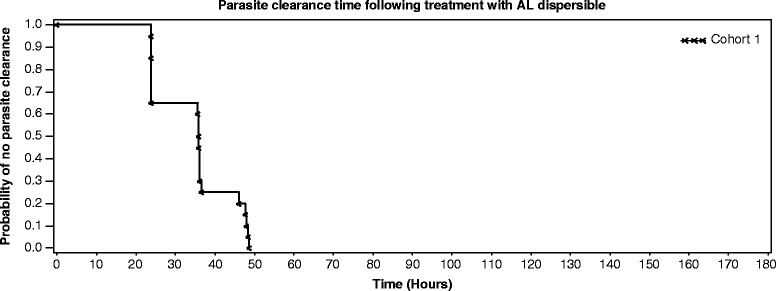
Parasite clearance time following treatment with artemether-lumefantrine dispersible. AL, artemether-lumefantrine. Parasite clearance time was defined as the time from first dose until total and continued disappearance of asexual forms for at least a further 48 hours (based on central microscopy reading). Subjects withdrawn from the study or on rescue medication before parasite clearance or those without parasite clearance till day 7 were considered censored at the time of withdrawal/start of rescue medication/day 7, whichever occurred earlier.

**Figure 3:**
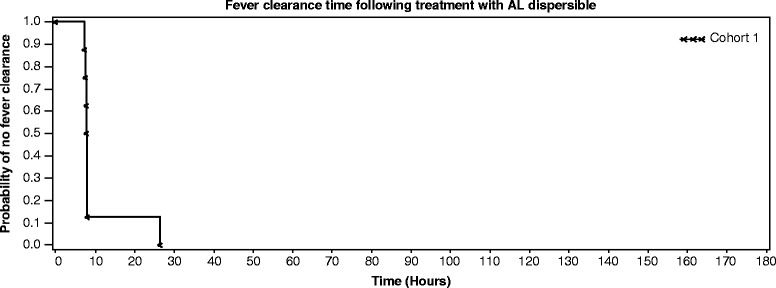
Fever clearance time following treatment with artemether-lumefantrine dispersible. AL, artemether–lumefantrine. Fever clearance time was defined as the time from first dose until the axillary temperature decreased and remained below 37.5^ο^C for at least a further 48 hours. Subjects who were withdrawn from the study or who received anti-malarial rescue medication, within seven days and before the clearance was achieved were considered censored at the time of withdrawal/start of rescue medication.

The PCR-corrected cure rate on days 14, 28 and 42 was 100% in the EPS and PPS populations (95% confidence interval (CI): 79.4-100), whereas it was 80% for the FAS population (95% CI: 56.3-94.3). Four patients did not have a microscopy reading after day 7 and were hence conservatively classified as treatment failures. The uncorrected cure rate on day 14 was also 100% (95% CI: 79.4-100; 79.4-100) in the EPS and PPS populations. However, the uncorrected cure rates were lower on days 28 and 42 in all populations (Table 2). The mean time to asexual parasite clearance was 29.1 ± 9.6 and 30.7 ± 8.8 hours in the FAS and EPS/PPS populations, respectively. Only one patient was infected with *Plasmodium ovale* in addition to *P. falciparum* at baseline, which persisted up to 72 hours and cleared. AL dispersible rapidly cleared fever with a mean time to fever clearance of 4.0 ± 6.4 and 4.1 ± 7.0 hours in the FAS and EPS/PPS populations, respectively. Complete (100%) asexual parasite clearance was achieved by 48 hours (Figure 2) and fever clearance by 26 hours (Figure 3). The mean gametocyte clearance time was 36.3 ± 77.3 and 43.9 ± 85.1 hours in the FAS and EPS/PPS populations, respectively. AL dispersible rapidly cleared gametocytes over time. By eight hours on day 1, gametocyte clearance was 90 and 87.5% in the FAS and EPS/PPS populations, respectively. After 24 hours, 20% of FAS and 25% of EPS/PPS populations had gametocytes, and by the end of 48 hours only one patient had gametocytes. Complete gametocyte clearance was observed by day 14 in all patients.

**Table 2:** **Parasitological cure rates on days 14, 28 and 42**

**Population**	**PCR-corrected cure rates**	**PCR-uncorrected cure rates**
	**n (%)**	**n (%)**
	**(95% CI)**	**(95% CI)**
**Day 14**		
FAS (N = 20)	16 (80)	16 (80)
	(53.6, 94.3)	(53.6, 94.3)
EPS (N = 16)	16 (100)	16 (100)
	(79.4, 100)	(79.4, 100)
PPS (N = 16)	16 (100)	16 (100)
	(79.4, 100)	(79.4, 100)
**Day 28**		
FAS (N = 20)	16 (80)	10 (50)
	(53.6, 94.3)	(27.2, 72.8)
EPS (N = 16)	16 (100)	10 (62.5)
	(79.4, 100)	(35.4, 84.8)
PPS (N = 16)	16 (100)	10 (62.5)
	(79.4, 100)	(35.4, 84.8)
**Day 42**		
FAS (N = 20)	16 (80)	7 (35)
	(53.6, 94.3)	(15.4, 59.2)
EPS (N = 16)	16 (100)	7 (43.8)
	(79.4, 100)	(19.8, 70.1)
PPS (N = 16)	16 (100)	7 (43.8)
	(79.4, 100)	(19.8, 70.1)

CI, Exact Pearson-Clopper two-sided 95% confidence limits; EPS, evaluable population set; FAS, full analysis set; PPS, per protocol set.

Parasitaemia results were taken from the central microscopy reading.

There were no deaths or SAEs reported during the six-week core follow-up period, and all AEs reported were mild to moderate in severity. At least one AE was reported by most patients (85%) during the core follow-up period. The most frequently reported AEs were new episodes of uncomplicated malaria (i.e., malaria re-infection) reported in 55% of patients during the follow-up period, followed by anaemia (35%; mild 5%, moderate 5%, severe 25%) and bronchitis (30%). Pyrexia, a manifestation of malaria, was also reported as an AE. Vomiting was reported as a drug-related AE in four (20%) patients (Table 3). One patient discontinued the study drug because of vomiting on day 1, which was moderate in intensity. However, this was not suspected to be related to the study drug and resolved by day 4. Out of the three SAEs reported during the long-term follow-up, two patients died and one patient was reported as having cerebral malaria and anaemia. The cerebral malaria resolved after appropriate treatment with quinine and antipyretics. A female patient aged 107 days died from acute diarrhoea with dehydration 52 days after completion of core follow-up (day 95), but had no prior AE or SAE record. Another patient, a 75-day-old male infant, died suddenly at home on an unrecorded date due to unknown etiology, after experiencing anorexia one day before death. Table 4 shows the proportion of patients with laboratory test abnormalities. Haemoglobin and blood chemistry shifts were mostly of grade 1 toxicity and no patients had worsening from normal to grade 3 or 4 toxicity.

**Table 3:** **Most common adverse events**

**Adverse event**	**N = 20**
	**n (%)**
Malaria	11 (55)
Anaemia	7 (35)
Bronchitis	6 (30)
Pyrexia	5 (25)
Vomiting	4 (20)
Gastroenteritis	2 (10)
Rhinitis	2 (10)

**Table 4:** **Patients with haematologic and blood chemistry abnormalities**

		**Day 3**	**Day 7**	**Day 14**	**Day 28**	**Day 42**
		**n (%)**	**n (%)**	**n (%)**	**n (%)**	**n (%)**
**Haematology parameters**						
Haemoglobin	Low	1 (6.3)	2 (10.5)	1 (8.3)	1 (12.5)	1 (25.0)
	High	0	0	0	0	0
Haematocrit	Low	1 (6.3)	1 (5.3)	1 (8.3)	1 (12.5)	0
	High	0	0	0	0	0
Neutrophils	Low	3 (18.8)	3 (17.6)	2 (20.0)	2 (25.0)	0
	High	0	1 (5.9)	0	0	1 (25.0)
Platelets	Low	1 (6.3)	1 (6.3)	1 (10.0)	1 (12.5)	0
	High	0	4 (25.0)	5 (50.0)	0	2 (50.0)
**Blood chemistry parameters**						
Bilirubin (total)	Low	1 (6.3)	0		0	0
	High	2 (12.5)	0		0	0
Creatinine	Low	3 (18.8)	3 (18.8)		0	0
	High	0	1 (6.3)		0	0
Glucose	Low	0	0		0	0
	High	0	1 (6.3)		0	0
SGOT	Low	0	0		0	0
	High	1 (6.7)	0		0	0
SGPT	Low	0	0		0	0
	High	0	0		0	0

SGOT, serum glutamic oxaloacetic transaminase; SGPT, serum glutamic pyruvic transaminase.

The mean ± SD score on the Shoklo neurodevelopmental scale for motor milestones (Section A) was 27.1 ± 4.5, coordination (Section B) 24.8 ± 4.6, tone (Section C) 16.6 ± 2.6, and behaviour (Section D) 14.4 ± 1.2. The overall total score mean ± SD (sum of A, B, C, and D) was 82.8 ± 9.4. The scores were within the normal range in all four domains of the scale.

**Figure 4:**
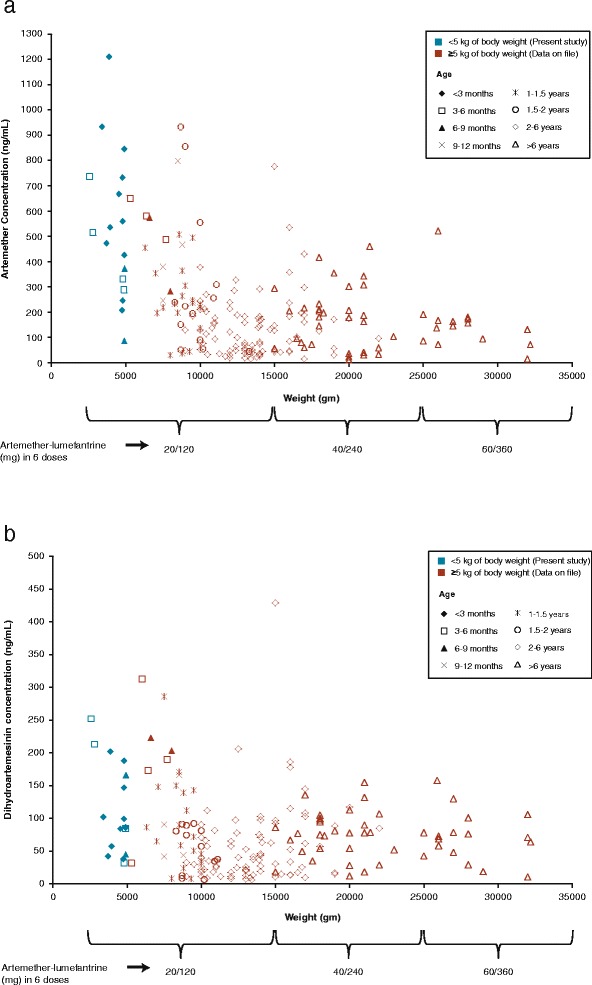
Comparison of artemether (**a**) and dihydroartemisinin (**b**) exposure in different age and body weight groups.

The mean artemether and DHA concentrations at one and two hours post-first dose were two- to three-fold greater compared with historical data in infants/children ≥5 kg (Table 5). Two patients had artemether concentrations higher than the range observed in infants/children ≥5 kg (Figure 4). Mean lumefantrine concentrations and range were similar to historical data in infants/children ≥5 kg except at day 7 where the mean lumefantrine concentration was found to be almost two-fold greater than values seen in infants ≥5 kg.

**Table 5:** **Pharmacokinetic exposure of artemether, dihydroartemisinin, and lumefantrine: comparison with historical data from older infants and children**

**Time point post-first dose**	**Current study**		**Historical data**	
	**Mean ± SD (CV %) [n]**	**Range**	**Mean ± SD (CV %) [n]**	**Range**
	**Artemether concentration (ng/mL)**			
**1 h**	446 ± 321 (72%) [15]	19.7-1210	139 ± 160 (116%) [173]	0-932
**2 h**	380 ± 262 (68%) [18]	0-933	140 ± 122 (87%) [170]	0-776
	**Dihydroartemisinin concentration (ng/mL)**			
**1 h**	87.6 ± 58.9 (67%) [15]	7.2-202	46.0 ± 54.2 (118%) [177]	0-345
**2 h**	93.4 ± 67.0 (72%) [18]	0-252	57.4 ± 57.5 (100%) [178]	0-429
	**Lumefantrine concentration (μg/mL)**			
**30 h**	No sample taken		5.29 ± 4.28 (81%) [63]	0-23.7
**54 h**	6.50 ± 3.37 (52%) [17]	0.819-12.5	5.84 ± 4.28 (73%) [62]	0.524-21.4
**66 h**	6.04 ± 2.77 (45%) [17]	2.27-12.8	6.98 ± 5.29 (76%) [323]	0.069-42.0
**84 h**	3.40 ± 2.28 (67%) [17]	1.16-8.72	3.02 ± 2.08 (69%) [49]	0.010-7.80
**168 h (Day 7)**	0.815 ± 0.567 (70%) [16]	0.200-2.39	0.386 ± 0.326 (84%) [63]	0-1.71
**336 h**	No sample taken		0.541 ± 2.13 (394%) [65]	0-12.2

CV, coefficient of variation; h, hour; SD, standard deviation.

## Discussion

In the current study that evaluated the efficacy, safety and PK of AL dispersible in infants aged >28 days and weighing <5 kg, AL dispersible was efficacious for the treatment of uncomplicated falciparum malaria. The PCR-corrected cure rate (100%) on days 28 and 42 for the EPS and PPS populations was slightly higher than earlier reports in infants and children ≥5 kg [4,12]. The uncorrected cure rates on days 28 and 42 reflect a high intensity of malaria transmission and high incidence of new infections in the study sub-region. The response to treatment was rapid in the study patients with faster mean time to parasite clearance and similar fever clearance to that reported earlier in children 5 to <10 kg of body weight [5,12].

AL dispersible was well tolerated with no new AEs reported. Except for one patient who developed cerebral malaria and anaemia in the long-term follow-up, all AEs were mild to moderate in intensity and the most commonly reported AEs were indicative of signs or symptoms of malaria, in line with previous reports in older infants and children [4,12]. As expected in this age group, vomiting was reported in four patients and was considered a drug-related AE, as in previous studies [4,5,13]. The two deaths reported in this study were reported after the core follow-up period and were deemed unrelated to study medication or to the index malaria episode. One patient had reported cerebral malaria and anaemia as SAEs during the long-term follow-up period, which resolved after treatment and were not considered to be related to study drug.

Artemether and DHA concentrations, on average, were found to be two- to three-fold greater in infants <5 kg as compared with historical levels [14] in older infants and children ≥5 kg, while those of lumefantrine appeared similar overall [4]. When combined with the data from infants and children ≥5 kg, a general trend of increasing exposure to artemether was observed with decreasing age and weight, especially in patients <10 kg and below the age of ~12 months (Figure 4). Thus, exposure to AL dispersible appears to be a complex function of both age and weight below the age of 12 months. Simple allometric scaling (based on relative body surface area and weight) suggests that artemether and lumefantrine doses (20 mg artemether and 120 mg lumefantrine twice daily for three days) used in this study are on the higher side for infants <5 kg. However, the available lumefantrine model from older children and adults cannot be used to derive a new dose recommendation for the fixed-dose combination of AL, because of an inherent complexity in predicting exposure in young infants and neonates [15]. Infants and neonates have different maturation stages for the absorption, distribution and disposition paths of artemether, DHA and lumefantrine, making predictions unreliable in this sub-population.

Artemether exposure was consistently high in infants <5 kg and aged less than three months, and infants with the lowest body weight and age had the highest exposure. Artemether undergoes a high first pass metabolism and is predominantly metabolized by the CYP3A4 enzyme [16]. CYP3A4 is present both in the gut and liver and is reported to have low activity in infants <12 months of age [15], particularly during the first week of life [17]. Hence, higher artemether exposure in infants could be primarily caused by immature CYP3A4 metabolism. Further decrease in the CYP3A4 activity in neonates ≤28 days would be expected to increase artemether exposure disproportionally.

DHA concentrations also increased, though to a lesser extent than artemether. This could result from the different metabolic pathways and the relatively lower first pass effect of DHA. DHA undergoes metabolism by glucuronidation, predominantly by UGT1A9 and UGT2B7, which are mostly hepatic. UGTs have decreased activity in newborns and young children as compared with adolescents and adults [15]. In neonates, there could be a significantly lower activity of these metabolic enzymes.

Given the known neurotoxic effects of artemisinins in animal studies [18-20], the neurologic safety of AL has been of interest in several human studies. Neurotoxicity has only been reported after intramuscular artemether administration in adult rats at 25 mg/kg/day for seven to14 days and in adult dogs at doses ≥20 mg/kg/day for eight days, but its relevance to neonates/infants is unknown. Although artemisinins have been known to cause neurotoxic effects in animals [18-20], there is no conclusive evidence of neurotoxic effects in adults or children [4,21]. Further, neurotoxicity has not been observed after oral artemether administration [22], suggesting that it is the total exposure over an extended period that leads to this toxicity, which can occur with intramuscular administration because of its slow continuous absorption without first pass effect. However, it was not possible to calculate reliable AUC in infants in this study because of the limitations in both the number and total volume of blood samples obtained from this population.

Higher exposures (C_max_ and AUC) and greater systemic toxicity with decreasing age of rat pups after artemether oral administration were reported [22]. Greater systemic toxicity was observed in the younger animals (mortality in seven to 13-day-old pups started at 30 mg/kg/day dose but no mortality in older pups was observed at this dose). In adult rats, neither mortality nor clinical neurotoxicity was observed at equivalent or higher artemether exposures for a longer duration than that in young rats. Therefore, there could be a concern that younger infants with a body weight <5 kg may be at particular risk for toxicity not observed in children or adults.

The age-appropriate Shoklo neurodevelopmental scale assessment that was performed at the long-term follow-up visit was selected as it focused on coordination and concentration, two areas where the neurological adverse effects of artemisinins are expected. The mean and median behaviour scores were close to the maximum possible value observed earlier [23]; the median to lower limit of range scores for coordination or behaviour milestones at 12 months were comparable to the Karen ethnic minority or the London cohorts [11]. The same variables were slightly higher for tone milestones in this study. This test was administered to children once, at the age of 12 months, in this study consisting of a single treatment arm. Thus, the present study cannot provide a baseline for comparison between different ages nor data from a comparative arm. Further, although the Shoklo scale was developed for countries with limited healthcare resources, the limitations of the present study mean that these results must be viewed with caution.

AL is a fixed-dose combination treatment, meaning that adjusting the dose of artemether by giving fractional doses of the dispersible tablet while maintaining that of lumefantrine unchanged is not feasible. The dispersible tablet neither has a break score nor is suitable for splitting, and the dispersion formed is not homogenous. Thus, half the quantity of artemether and lumefantrine cannot be guaranteed with approximately half of a dispersible tablet or half an aliquot of the dispersion. Dose adjustment with one dispersible tablet administered once daily for three days may also not reduce the risk of potential artemether and DHA toxicity as the C_max_ values would remain elevated. Lumefantrine exposure is the principal determinant of AL anti-malarial activity [24], which drives the 28-day cure rate. A decrease in lumefantrine dose and exposure by using a fractional dose might put the patients at risk of treatment failure. Thus, on this basis, clear dosing recommendations cannot be made in this population of neonates and infants weighing <5 kg.

## Conclusion

The three-day regimen of the dispersible tablet formulation of AL was similar in efficacy to that in older infants and was well tolerated for uncomplicated falciparum malaria treatment in infants <5 kg of body weight and aged >28 days, with no new safety findings. On average, artemether and DHA (but not lumefantrine) systemic exposures were two- to three-fold greater for patients in this study as compared to infants and children ≥5 kg. Although no neurotoxicity has been reported after oral doses, implications for potential toxicity in infants and neonates are unknown. Therefore, based on the results of the current study, clear dosing recommendations cannot be made in infants <5 kg. Furthermore, the findings from this study underscore the need for detailed characterization of the pharmacokinetic/pharmacodynamic and safety profile of ACT and other anti-malarial drugs for use in this population to determine optimal doses rather than relying on empirical doses.
